# The concept and treatment of psychological trauma

**DOI:** 10.3402/ejpt.v5.26514

**Published:** 2014-12-09

**Authors:** Harold Kudler

**Affiliations:** Department of Psychiatry and Behavioral Sciences, Duke University, Durham, NC, USA

**Keywords:** psychological trauma, PTSD, theory, treatment, diagnosis, evidence-based interventions, transference, countertransference

## Abstract

Despite a large and rapidly expanding literature on psychological trauma, many fundamental questions remain about its basic nature: Is it a psychological problem or a biological one?; Is it a past event somehow stuck in the present or is it something new which has been triggered and shaped by that event?; Does it reside only within the patient or does it live between the patient and other people (including within the therapeutic relationship)? This presentation will review the history of the concept of psychological trauma and explore the theoretical bases for current evidence-based psychotherapies for PTSD, each of which will be shown to describe psychological trauma as a problem in bringing the past and the present together in memory and cognition. These theories primarily differ on the question of whether a traumatic memory becomes pathogenic, because it cannot be biologically processed or because it must be psychologically avoided. Psychoanalytic concepts of transference and countertransference will be shown to be of practical importance regardless of the type of treatment chosen. If researchers and clinicians can build on what they hold in common rather than become divided by their differences, we can improve our ability to understand and alleviate the effects of psychological trauma.

